# Development and validation of nomogram with tumor microenvironment-related genes and clinical factors for predicting overall survival of endometrial cancer

**DOI:** 10.7150/jca.51493

**Published:** 2021-04-23

**Authors:** Qian Chen, Shu Wang, Jing-He Lang

**Affiliations:** Department of Gynecology and Obstetrics, Peking Union Medical College Hospital (PUMCH), Chinese Academy of Medical Sciences (CAMS) & Peking Union Medical College, National Clinical Research Center for Obstetric & Gynecologic Diseases. Beijing, P.R, China

**Keywords:** endometrial cancer, tumor microenvironment, nomogram, prognosis, immune infiltration.

## Abstract

**Background:** Tumor microenvironment (TME) has attracted lots of attention with its important role in the tumor development. This study aimed to explore TME- related genes of prognostic value in patients with endometrial cancer (EC) and establish a prediction model for EC.

**Methods:** The RNA-Seq data and clinicopathological characteristics of 521 subjects were obtained from The Cancer Genome Atlas (TCGA) database. Differentially expressed genes (DEGs) were identified based on the immune and stromal scores, which were calculated by the ESTIMATE algorithm. Hub genes were initially screened using the Cytoscape and further selected through Cox regression. Gene correlation analysis was performed in TIMER database. A nomogram was constructed integrating prognosis-related hub genes and clinical factors and validated in the validation group. Risk stratification was performed based on the nomogram.

**Results:** Three TME-related hub genes (*CCR5*, *FCER1G,* and* ITGB2*) were found with significant prognostic value for EC patients. The expression of *CCR5*, *FCER1G,* and* ITGB2* were significantly correlated with various immune cells infiltration. Based on the Cox regression, a nomogram was constructed by integrating five predictors (stage, grade, immune score, expression of *FCER1G*, and *ITGB2*), with a C-index of 0.765. Discrimination of the model was confirmed in the validation group (C-index: 0.716). The calibration curves for the 3- and 5- year survival indicated good calibration. Patients in high- and low- risk groups presented significantly different survival outcomes (P<0.001) in both discovery and validation group.

**Conclusion:** TME-related hub genes of prognostic value identified in our study may provide references for the mechanisms underlying EC development and the immunotherapy for EC. The prediction model may help assess the prognosis of EC patients.

## Introduction

Endometrial cancer (EC) is one of the most frequently diagnosed cancers in female genital tract. According to the data from the National Central Cancer Registry of China (NCCR), the number of new cases and deaths due to EC in 2015 was about 63400 and 21800, respectively 1. Furthermore, the increasing incidence of EC is predicted to continue in the next few decades.

The majority of EC patients are diagnosed at early stage with a good prognosis, while a small part of patients are faced with a dramatically decreased five-year survival due to the progression of EC 2. Mechanisms underlying the progression and metastasis of EC is still poorly understood. Typically, EC is divided into 2 categories: type I and type II, which is widely used in clinical work 3. In 2013, a new classification of EC was proposed based on data from The Cancer Genome Atlas Research Network (TCGA): ultramutated/polymerase ε (POLE)-mutated; hypermutated/MSI (MSI-H); copy number-low (microsatellite stable [MSS]) and copy number-high 4. With well-described molecular characteristics of EC, this classification may carry important advantages in tailoring personalized treatments.

In addition to the molecular heterogeneity, recent researches have also shed light on the role of tumor microenvironment (TME) in EC progression. TME is a complex and dynamic system consisting of immune cells, stromal cells, extracellular matrix, and a variety of cytokines and chemokines, which presents close connection with tumor cells 5. The reciprocal interactions between EC cells and components of TME create a more appropriate environment to facilitate the development of EC 6. For example, tumor immune escape has been proven to be an important strategy for tumor progression, which involves the process of immune suppression 7. More and more clinical trials have been carried out to investigate the effect of PD-L1 antagonists, a kind of immune checkpoint blockers aiming to restore immune system function, in advanced EC patients 8.

A sufficient understanding of TME profile is preponderant to further investigate the mechanism of cancer development. ESTIMATE algorithm is a method created to predict tumor purity and infiltration of immune/stromal cells in tumor tissues based on molecular expression data 9. Studies published have reported the value of immune/stromal scores calculated with ESTIMATE algorithm in prognosis assessment of renal cell cancer and breast cancer 10, 11, while this approach has not been evaluated in EC patients.

In the present study, we obtained immune/stromal scores of EC patients from The Cancer Genome Atlas (TCGA) database through ESTIMATE algorithm and identified multiple TME-related genes with prognostic implications. Functional analysis and immune infiltration correlations were performed for selected hub genes to investigate their potential value in the mechanism of EC progression and possible application for immune therapy. Furthermore, we integrated the clinical parameters and TME-related genes to construct prediction model to facilitate the management of EC patients in the clinical work.

## Methods

### 1. Data collection from TCGA

The data of RNA‐seq expression and corresponding clinical information of EC patients were collected from The Cancer Genome Atlas (TCGA) online database (http://cancergenome.nih.gov).

Clinical characteristics including age, stage, grade, body mass index (BMI), histological type, survival status, and survival time were extracted. Patients with primary tumor site of corpus uteri were included. The exclusion criteria were:1) Patients lack of information on survival outcomes; 2) Patients with a survival time of less than 30 days.

Totally, 521 patients were included and randomly divided into a discovery group (n=261) and a validation group (n=260), which were used to identify hub genes and to verify the hub genes respectively.

### 2. Analysis with ESTIMATE algorithm

After preliminary data processing, genes were eliminated using “limma” package in R version 3.6.0 (http://www.r-project.org) if the expression values were “zero”. The immune, stromal and ESTIMATE score were calculated based on the expression data using “estimate” package (http://r-forge.rproject.org; repos=rforge, dependencies=TRUE) 9.

### 3. Clinical relevance evaluation and survival analysis

Clinical characters including stage and grade of the whole EC cohort were analyzed on the basis of immune, stromal and ESTIMATE score. Kaplan-Meier method (Log-rank test) was applied to analyze the prognostic value of the three kinds of scores in R (“survival” package).

### 4. Identification and functional analysis of differentially expressed genes

Patients were divided into two subgroups using the median value of the scores as the cut-off value, respectively. In the discovery group, the package of “limma” in R was used to identify the differentially expressed genes (DEGs) between high- and low-immune score samples, high- and low-stromal score samples, respectively. The criteria of |log2fold change (log2FC)| >1 and adjusted P-value <0.05 was set as the threshold to screen DEGs. Unsupervised hierarchical clustering was then performed using “pheatmap” function in R. The overlapping ones of the two sets of DEGs (immune set and stromal set) were selected as TME-related genes and further analyzed.

Gene ontology (GO) analysis including the cellular component(CC), molecular function(MF), and biological process(BP) and Kyoto Encyclopedia of Genes and Genomes (KEGG) pathway enrichment analysis were conducted based on the TME-related genes using “clusterProfiler” package in R. P<0.05 was considered statistically significant.

### 5. Protein-protein interaction (PPI) network and hub genes selection

We applied the Search Tool for the Retrieval of Interacting Genes (STRING) database (https://string-db.org/)(Version 11.0) to evaluate the interactive relationships among TME-related genes, with a combined score>0.7 (medium confidence). PPI networks were then constructed using the Cytoscape software(Version 3.6.1). A plug-in of Cytoscape, Molecular Complex Detection (MCODE), was used to cluster and screen modules within PPI network with MCODE score >5 and number of nodes >5. Another plugin, cytoHubba, was used to select hub genes. Visualization of functional enrichment of hub genes was analyzed using the Cytoscape plugin of Cluego and Cluepedia.

### 6. Hub Genes of prognostic value and gene correlation analysis in TIMER database

A Cox proportional hazards regression analysis was performed to explore the prognostic value of the hub genes, and those of significant prognostic value (P<0.05) were selected for further analysis.

The correlation of expression of selected genes with the abundance of immune infiltrates, including B cells, CD4+ T cells, CD8+ T cells, neutrophils, macrophages, and dendritic cells were analyzed in TIMER database (https://cistrome.shinyapps.io/timer/). Spearman's correlation and statistical significance were applied to assess the correlation. P-values <0.05 was considered statistically significant 12.

### 7. Nomogram construction with hub genes and clinical factors

In this part, we reclassified patients with stage I and stage II EC as early-stage patients, and patients with stage III and stage IV EC as early-stage patients. G1 and G2 EC were defined as low-grade EC, and others were defined as high-grade EC. Moreover, we adopted the median values of immune/stromal/ESTIMATE score as the cutoff value.

Prognostic clinical factors (P<0.05) were selected through the univariate Cox regression model. The multivariate Cox proportional hazard regression analysis was performed on the selected hub genes and clinical parameters. A prognostic nomogram was developed incorporating factors identified based on the Akaike information criterion (AIC) to predict 3‐ and 5‐year overall survival (OS). The nomogram was created with R package of “rms”.

### 8. Validation of prediction model

The prediction model was validated in terms of internal (the discovery group) and external (the validation group) discrimination and calibration assessments. The concordance index (C-index) was calculated to quantitatively assess the discriminative ability. The calibration curves were plotted to evaluate the agreement between model‐predicted and actual survival using the “rms” package of R software.

### 9. Risk stratification of EC patients

A risk score was obtained from the nomogram for each patient. Patients in the discovery and validation group were divided into high- and low-risk groups based on the risk scores, respectively. Kaplan-Meier method and log-rank test were applied to evaluate the difference in the survival curves of the high- and low-risk groups.

## Result

### 1. Patient characteristics of the cohort

Relative information of patients included in our study was illustrated in Table 1. The median age of the whole cohort was 64.0 (range, 31-89) years. Patients in stage I accounted for 62.4% (325/521), stage II 9.8% (51/521), stage III 22.6% (118/521) and stage IV 5.2% (27/521). We did not observe significant differences in the distribution of age, BMI, histological type, stage and grade between the discovery and the validation group. The median values of immune/stromal score were 219.94 (-1359.51 to 3614.68) and -808.41 (-2224.62 to 860.43), respectively.

**Figure 1:**
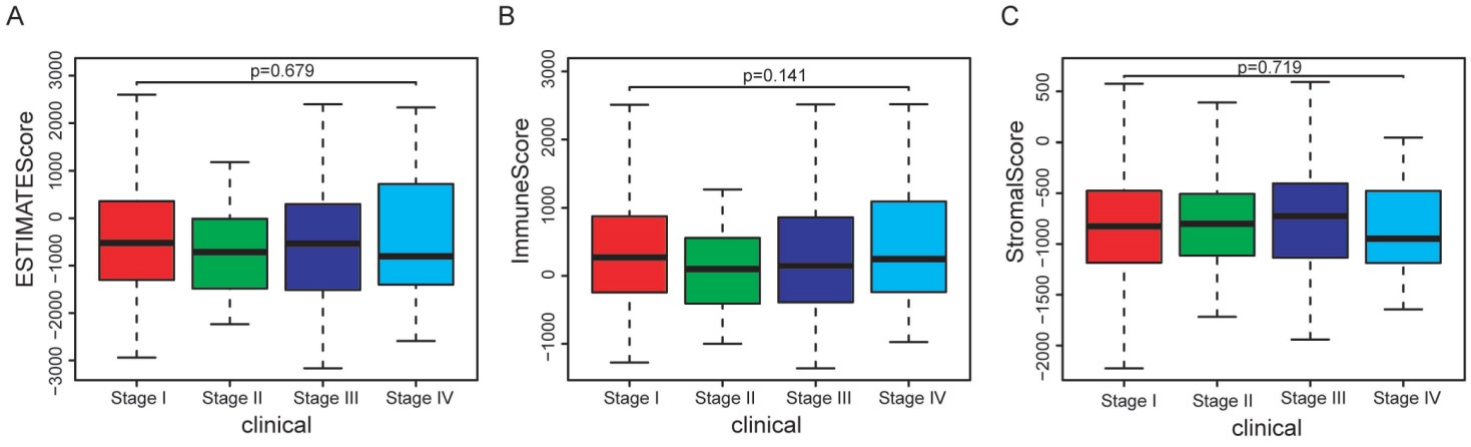
Association between immune/stromal/Estimate score and clinical features of EC patients. (A-C): No significant relationship was noticed between immune/stromal/Estimate score and stage. (D-F): The immune/stromal/Estimate score were not significantly related with grade.

**Figure 2:**
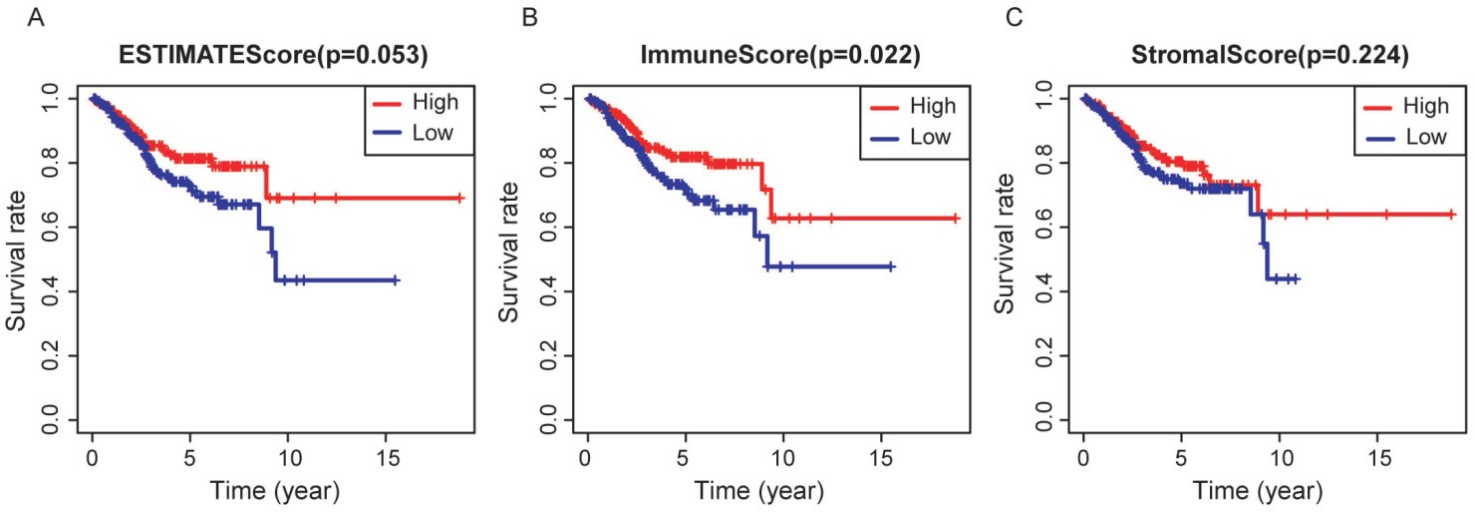
Association between immune/stromal/Estimate score and prognosis of EC patients. Elevated immune score was significantly associated with longer overall survival (p=0.022), while relations between stromal/Estimate score and OS were both not significant.

In the whole cohort, no significant association was observed between the stage of EC patients and the immune/stromal/ESTIMATE score (Figure 1). For the survival outcomes, increased immune sore (P=0.022) was significantly associated with longer OS, while the relationship between the stromal score (P=0.224) or ESTIMATE score (P=0.053) and OS were not significant (Figure 2).

### 2. Identification of DEGs based on immune and stromal score and functional enrichment analysis

**Figure 3:**
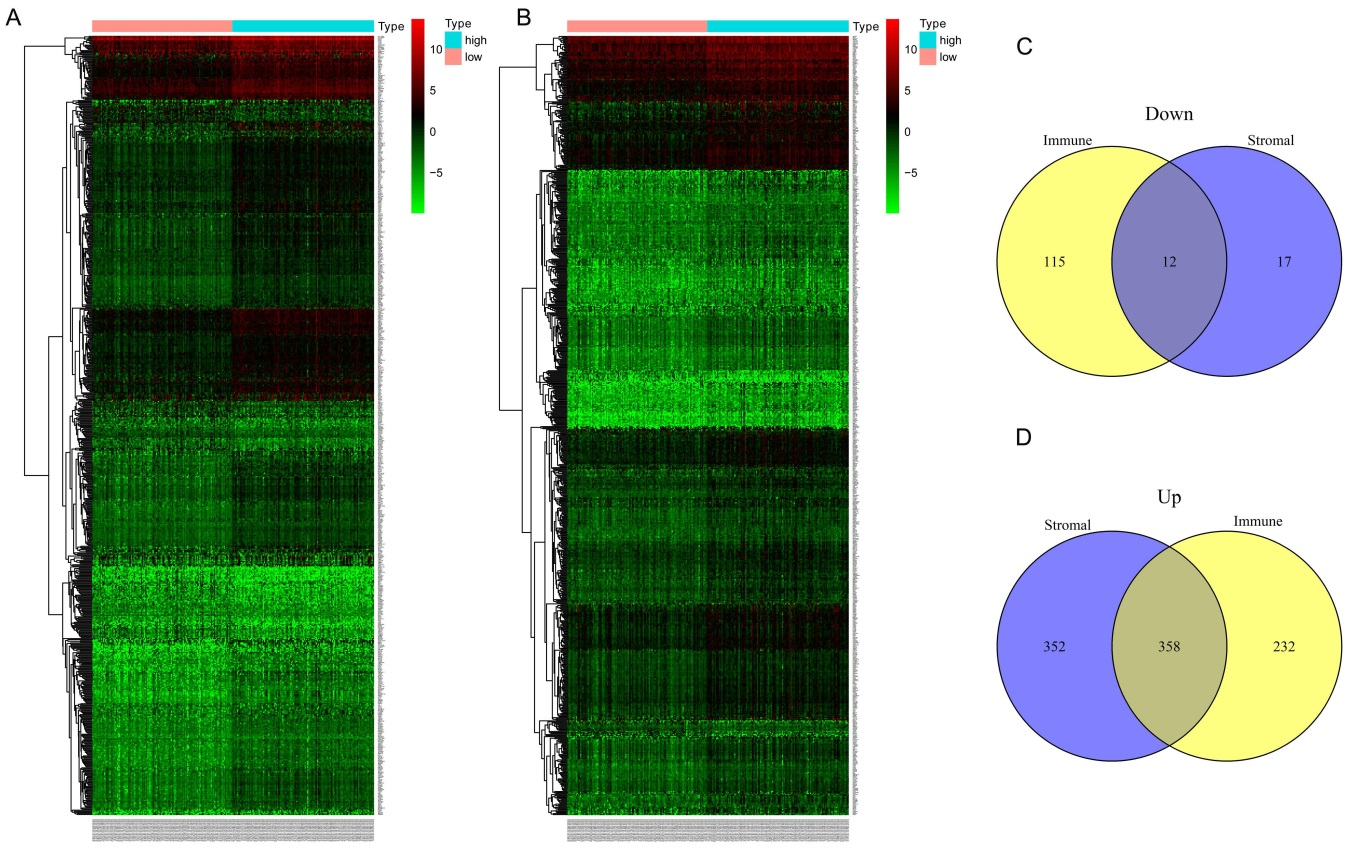
Differential expressed genes (DEGs)based on immune and stromal score. (A) Heat map of DEGs based on immune score comparison (high score vs. low score). (B) Heat map of DEGs based on stromal score comparison (high score vs. low score). (C-D) 12 common down-regulated genes and 358 common up-regulated genes identified in Venn diagrams.

In the discovery group, after comparison between the high-immune score group and low- immune score group, we obtained 590 up-regulated genes and 127 down-regulated genes (Figure 3A). Similarly, 710 up-regulated genes and 29 down-regulated genes were identified when we compared the high- stromal score group with low- stromal score group (Figure 3D). Finally, 370 common DEGs were obtained for the following analysis, of which 358 genes were up-regulated and 12 genes were down-regulated (Figure 3. C-D).

**Figure 4:**
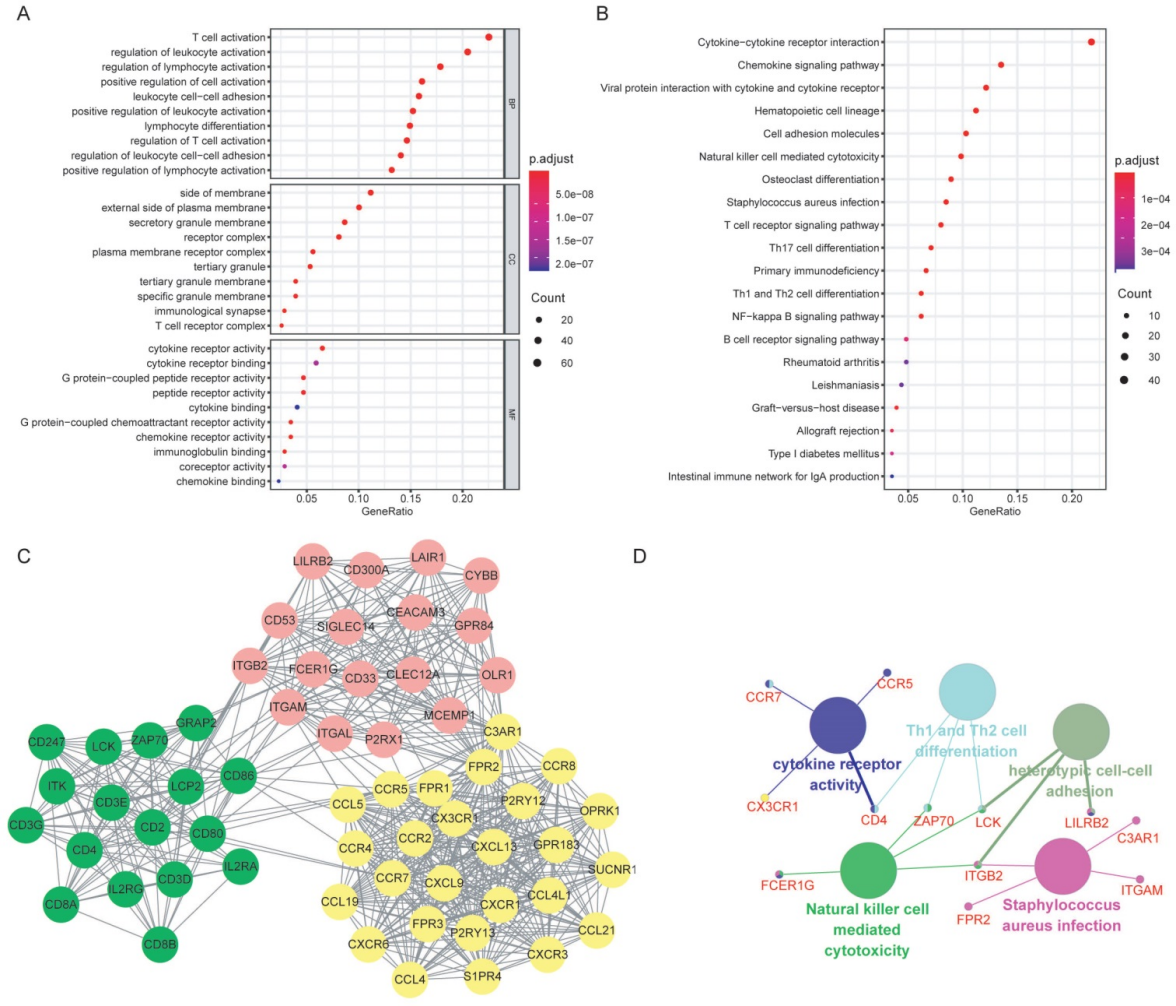
Functional enrichment analysis of the DEGs and the hub genes. (A) GO annotations of the DEGs: the top 10 enriched GO terms for BP, CC and MF, respectively. (B) KEGG pathway enrichment analysis of the DEGs. (C) PPI network was constructed based on the DEGs. Three significant clusters (in different color) were presented using MCODE, a plug-in of Cytoscape. (D)Functional enrichment analysis of the hub genes: the large nodes represented the GO or KEGG terms, and the small nodes were hub genes. Abbreviations: DEGs: differentially expressed genes; GO: Gene Ontology; KEGG: Kyoto Encyclopedia of Genes and Genomes; BP: biological process; CC: cellular component; MF: molecular function; PPI: protein-protein interaction.

GO analysis was performed for the 370 selected DEGs, with the top 10 function annotations of each term presented in Figure 4A. Most of DEGs were enriched in BPs, such as T cell activation, regulation of leukocyte and lymphocyte activation. Significant CC annotations (including side of membrane and external side of plasma membrane) and MF annotations (including cytokine receptor activity and binding) were also observed. KEGG pathway analysis revealed several significant pathways, such as cytokine-cytokine receptor interaction and chemokine signaling pathway (Figure 4B).

### 3. PPI network analysis and hub genes selection

The potential relationships among DEGs were explored via the online software STRING with a combined interaction score of > 0.7. After the analysis with MCODE in Cytoscape, we identified three clusters (presented in different color in Figure 4C) according to the criteria of MCODE score >5 and number of nodes >5. Thirteen hub genes were selected using the plug-in of cytoHubba: *C3AR1, CCR5, CCR7, FPR2, CD4, CD53, CX3CR1, FCER1G, ITGAM, ITGB2, LCK, LILRB2 and ZAP70*, all of which were up-regulated. Further functional annotations revealed that the hub genes mainly involved in cytokine receptor activity, Th1 and Th2 cell differentiation, heterotypic cell-cell adhesion, natural killer cell mediated cytotoxicity and staphylococcus aureus infection (Figure 4D).

### 4. Selection of prognosis-related hub genes and gene correlation analysis

Cox regression analysis was performed on the 13 selected hub genes based on the mRNA expression profile. It is indicated that elevated* CCR5* (P=0.027) and *ITGB2* (P=0.031) expression were significantly related with poorer prognosis, while elevated* FCER1G* (P=0.022) expression was associated with better prognosis.

**Figure 5:**
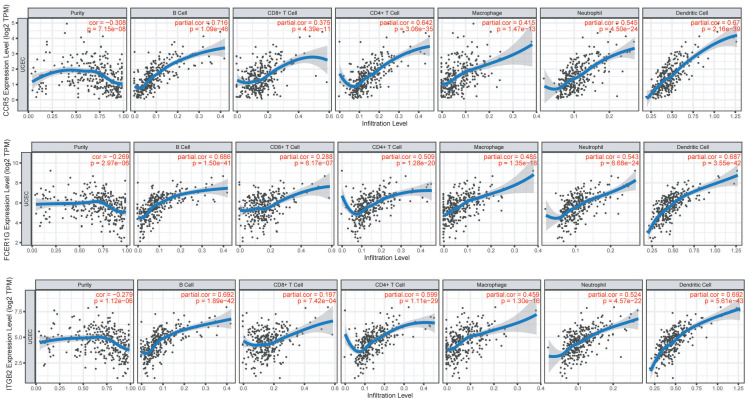
Immune infiltration of *CCR5*, *FCER1G,* and* ITGB2* in the TIMER database. The expression of *CCR5*, *FCER1G,* and* ITGB2* all presented significant positive correlations with infiltrating levels of B cell, CD8+ T cells, CD4+ T cells, macrophages, neutrophils, and dendritic cells in endometrial cancer.

We investigated the correlations of *CCR5*, *FCER1G* and *ITGB2* with immune infiltration levels in EC from TIMER (Figure 5). We observed that *CCR5* (cor=-0.3, P=1.63e-07), *FCER1G* (cor=-0.269, P=2.97e-06) and *ITGB2* (cor=-0.279, P=1.12e-06) were all significantly correlated with tumor purity in a negative manner. Increased expression of these three genes were significantly associated with increased infiltration levels of B cell, CD8+ T cell, CD8+ T cell, macrophage, neotrophil, and dendritic cell(P<0.01).

### 5. Nomogram construction and validation

**Figure 6:**
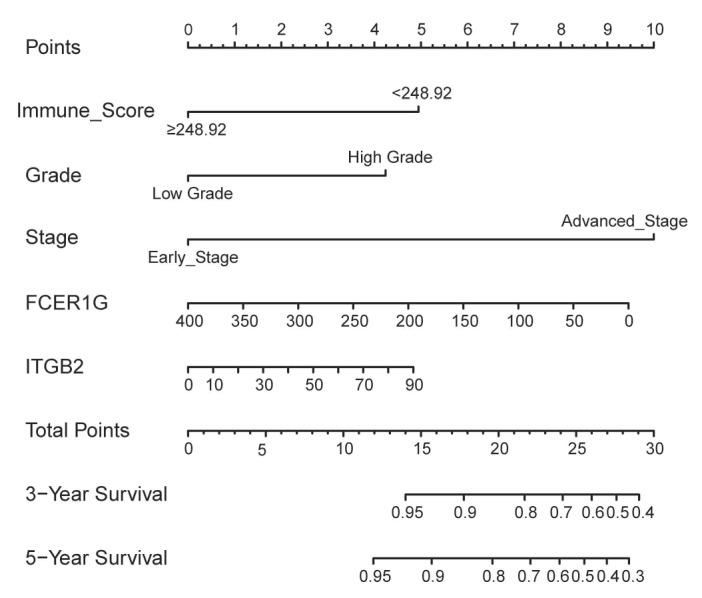
Nomogram to predict 3- and 5-year survival of patients with endometrial cancer.

In addition to the above three genes, stage (Hazard Ratio [HR]:2.746; P<0.001), grade (HR:3.214; P=0.006), histological type (HR:2.558; P=0.005) and immune score (HR:0.472; P=0.034) were significantly related with OS. Based on the AIC in the multivariate Cox regression analysis, stage, grade, immune score, expression of *FCER1G* and *ITGB2* were finally selected (AIC = 327.66) (Table 2). A nomogram was constructed by integrating these five predictors (Figure 6).

**Figure 7:**
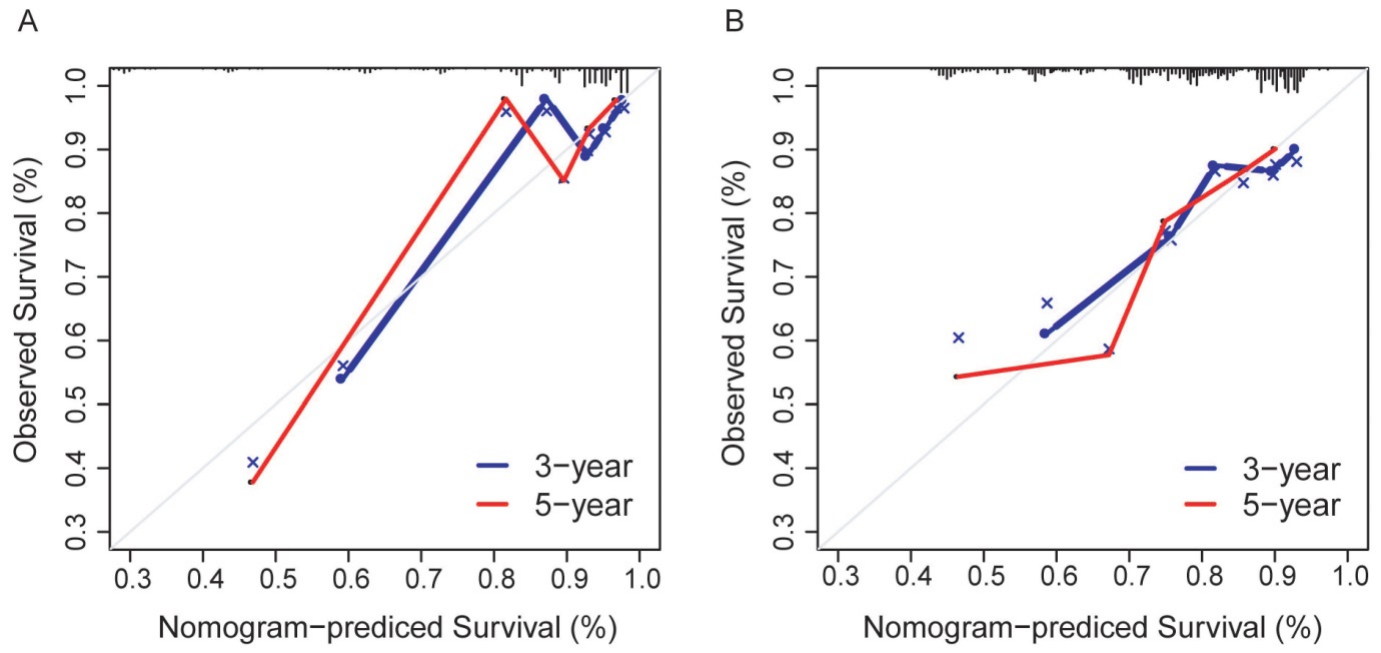
Calibration curves for the nomogram. (A) The discovery group; (B) The validation group.

The prediction model exhibited favorable discrimination with a C-index of 0.765 [95% confidence interval (CI), 0.676-0.855) in the discovery group, and a C-index of 0.716 (95% CI, 0.641-0.791) in the validation group. The calibration curves for the 3- and 5- year OS indicated good calibration in both discovery and validation group (Figure 7).

### 6. Risk stratification of EC patients

**Figure 8:**
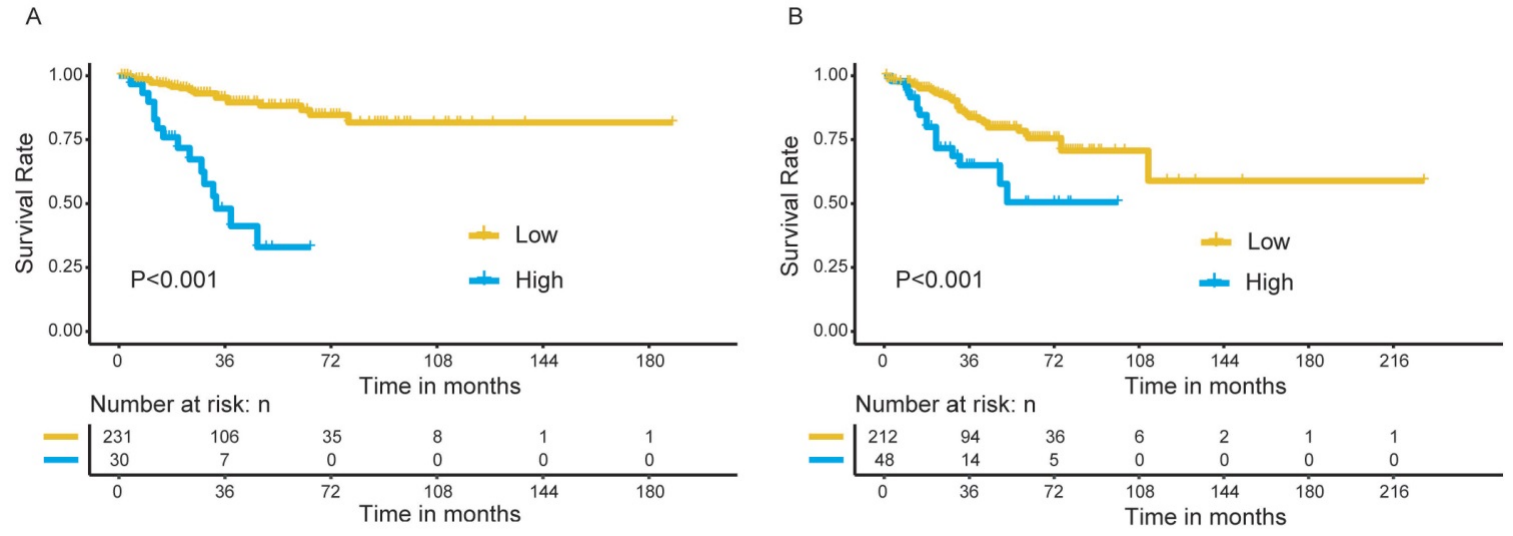
Survival analysis for patients in high- and low-risk group. (A) The discovery group; (B) The validation group.

All patients were stratified into high- and low-risk groups based on the risk scores obtained from the nomogram, with a total point of 24 as the cutoff value. We observed a significant difference in OS between the high- and low-risk patients in the discovery group (Figure 8A), which was further confirmed in the validation group (Figure 8B).

## Discussion

In the present study, we calculated the immune/stromal/ESTIMATE score using mRNA expression data of 521 EC patients collected from TCGA database. A total of 370 overlapping DEGs were identified in the discovery group based on the immune and stromal scores. Of the thirteen TME-related hub genes, *CCR5*, *FCER1G,* and *ITGB2* were found with significant prognostic value for EC patients. A nomogram was constructed by integrating five predictors (stage, grade, immune score, expression of* FCER1G,* and *ITGB2*), which were identified based on the AIC. The prediction model presented favorable discrimination and calibration in the discovery and validation group.

The immune score was significantly associated with survival outcomes of EC patients in our study, with higher score indicating longer overall survival time. Tumor-infiltrating lymphocytes (TIL), the important component of the TME, were reported to be associated with various types of cancer 13-15. Tumor-infiltrating T-lymphocytes presented higher specific immunological reactivity against tumor cells, some of which (such as cytotoxic T-lymphocytes, regulatory T-cells and memory T-lymphocytes) have been found to have impacts on prognosis of EC patients 16-20.

We included the stromal part when identifying DEGs given its essential role in tumor development 21, 22, although no significant relationship between stromal score and EC survival outcomes was observed. Functional analysis with the method of GO and KEGG as well as the construction of PPI further indicated the possible activities and interactions occurring in TME. *CCR5*, *FCER1G,* and* ITGB2* were then selected as prognosis-related hub genes for the following analysis. In another study, immune-related genes including *CD8*, *GZMA* and *HLA-A* were identified as favorable prognostic factors for EC patients 23.

CCR5, C-C chemokine receptor type 5, belongs to the 7-transmembrane G protein-coupled receptor family, with various ligands including CCL3 (MIP1a), CCL5 (RANTES) and so on 24. Published studies have revealed several important roles of CCR5 in the tumor prognosis, such as promoting invasion and metastasis of cancer, affecting immune responses and inducing resistance to cell death 25. Overexpressed CCR5 or the ligand CCL5 was reported to be related with poor prognosis of multiple cancers, such as breast cancer 26, ovarian cancer 27 and cervical cancer 28, which was in line with our results. Clinical trials are carrying out to target CCR5 for metastatic cancer 25.

As a constitutive component of the high-affinity immunoglobulin E (IgE) receptor and interleukin-3 receptor complex, the Fc epsilon receptor *FCER1G* involved in various signaling pathways in immune response^29^, ^30^. Fu et al. reported that high expression of *FCER1G* indicated favorable survival outcomes in patients with multiple myeloma, which was similar to our results 31. However, another study identified negative relationship between *FCER1G* and tumor progression. They also supported the simulative role of *FCER1G* in carcinogenesis, which was associated with immune reactions 32.

*ITGB2* is a leukocyte-specific integrin that participates in leukocyte adhesion to endothelium and the ensuing extravasation 33. In the current study, *ITGB2* appeared as a protective factor for EC patients. According to the study from Liu et al., the induction of* ITGB2* expression by Yes-associated protein (YAP) facilitates the invasion of cancer cells through the endothelium 34. The role of *ITGB2* in the tumorigenesis and the tumor metastasis needs further investigation.

The immune infiltration analysis of *CCR5*, *FCER1G,* and* ITGB2* revealed significantly positive correlations between their expression and the infiltration levels of B cells, CD8+ T cells, CD4+ T cells, macrophages, neutrophils, and dendritic cells, implicating the role of these three genes in regulating tumor immunology in EC. The relationship may provide references for mechanism studies in the future.

The emergency of immunotherapy has casted new light on antitumor treatment especially for advanced patients 35. Investigations on the compositions of TME and regulatory mechanisms are of importance to explore new targets for the treatment and create more effective immunotherapeutic strategies 6, 8, 36. Our study may offer ideas to better understand the TME profiles of EC patients. Furthermore, we combined the TME-related genes and the clinical parameters to build a prediction model, which may help to precisely estimate the prognosis of patients with EC.

We acknowledge that limitations exist in the present study. Analyses here were based on data from public databases. Subsequent experimental exploration and verification with external data from other medical centers are required, before translating our results into clinical work.

## Conclusions

In summary, after the analysis of immune and stromal score with data collected from TCGA database, we identified three TME-related hub genes, *CCR5*, *FCER1G,* and* ITGB2,* which are significantly related to survival outcomes of EC patients. A prediction model with TME-related genes and clinical factors was developed to help assess the prognosis of EC patients. The immune infiltration analysis of *CCR5*, *FCER1G,* and* ITGB2* indicated their roles in regulating tumor immunology in EC.

## Figures and Tables

**Table 1:** Baseline clinicopathological characteristics of the patients.

Variable	Discovery Group (N=261)	Validation Group (N=260)	P value
Age, y Median (range)	64.0 (34-88)	63.5 (31-89)	0.460
BMI, n, %			0.900
<30	105 (40.2)	106 (40.8)	
≥30	156 (59.8)	154 (59.2)	
Stage, n, %			0.368
Stage I	169 (64.8)	156 (60.0)	
Stage II	28 (10.7)	23 (8.8)	
Stage III	53 (20.3)	65 (25.0)	
Stage IV	11 (4.2)	16 (6.2)	
Grade, n, %			0.704
G1	47 (18.1)	48 (18.5)	
G2	62 (23.8)	54 (20.8)	
G3	148 (56.8)	151 (58.1)	
High-Grade	4 (1.5)	7 (2.7)	
Histological type, n, %			0.859
EEC	195 (74.7)	196 (75.4)	
Non-EEC	66 (25.3)	64 (24.6)	

EEC: endometrial endometrioid carcinoma.

**Table 2:** Multivariate Cox regression analysis based on the Akaike information criterion in the discovery group.

Variable		HR (95%CI)	P value
Stage			<0.001
	Early stage	Reference	
	Advanced stage	6.797 (3.3072-13.9701)	
Grade			0.058
	Low grade	Reference	
	High grade	2.254 (0.9736-5.2159)	
Immune score			0.009
	< 248.92	Reference	
	≥ 248.92	0.388 (0.191-0.788)	
FCER1G	continuous	0.996 (0.974-1.018)	0.685
ITGB2	continuous	1.010 (0.9416-1.0841)	0.774

HR: hazard ratio; CI: confidence interval.
